# Comparative analysis of fatigue and mental health in sexual and gender minority cancer survivors

**DOI:** 10.1007/s00520-026-10959-6

**Published:** 2026-07-08

**Authors:** Alique G. Topalian, Brad R. Woodie, Anne Fleischer, Shanna D. Stryker

**Affiliations:** 1 Department of Family and Community Medicine, Division of Cancer Survivorship and Supportive Services, University of Cincinnati College of Medicine, 231 Albert Sabin Way, Cincinnati, OH 45267 USA; 2 University of Cincinnati Cancer Center, the University of Cincinnati College of Medicine, Cincinnati, OH 45267 USA; 3 College of Medicine, University of Cincinnati College of Medicine, Cincinnati, OH 45267 USA; 4 Department of Rehabilitation, Exercise, and Nutrition Sciences, University of Cincinnati College of Allied Health, Cincinnati, OH USA

**Keywords:** Cancer survivors, Cancer-related fatigue, Sexual and gender minority, Mental health, LGBT

## Abstract

**Purpose:**

This study aims to compare self-reported fatigue and mental health in sexual and gender minority (SGM) cancer survivors and cisgender-heterosexual cancer survivors.

**Methods:**

Using data from the National Institutes of Health’s *All of Us R*esearch Program, survey responses from 36,684 respondents with a history of cancer were analyzed. Information collected from respondents included sexual orientation, gender, race, ethnicity, age at first cancer, history of anxiety or depression, and history of fatigue-related diagnoses. Outcomes included surveys assessing fatigue and mental health. SGM individuals were compared with majority groups using multivariable logistic regression.

**Results:**

SGM survivors represented 6.6% of the sample. SGM survivors had increased odds of high fatigue than cisgender-heterosexual survivors (OR = 1.33, 95% CI[1.17, 1.51], *p* < .001). SGM survivors also experienced higher odds of self-reported poor mental health when compared with cisgender-heterosexual survivors (OR = 1.85, 95% CI [1.64, 2.03], *p* < .001). After controlling for anxiety, depression, and fatigue, SGM cancer survivors had higher odds of self-rated poor or fair mental health compared to cisgender-heterosexual cancer survivors.

**Conclusions:**

Fatigue and mental health are worse among SGM cancer survivors compared to cisgender-heterosexual survivors. Future interventional studies are needed to mitigate the unique fatigue and mental health needs of this population.

**Implications:**

Given the higher burden of fatigue among SGM cancer survivors, cancer care providers should screen for and address fatigue and mental health needs of SGM individuals impacted by cancer before, during, and after treatment to minimize the impact on their daily life. Outcomes for SGM survivors could be improved by routine sexual orientation and gender identity data collection in oncology settings and cancer databases, by education for SGM-serving primary care clinicians outside of cancer care settings on the care needs of survivors, and by piloting SGM-tailored interventions to address fatigue.

## Background

Due to advances in cancer treatment and earlier detection, the number of cancer survivors is steadily increasing [1]. By 2030, the number of survivors in the United States is projected to grow to more than 22.1 million [2]. Due to cancer survivors living longer, there is a greater focus on managing the effects of cancer and its treatment throughout the lifespan [3, 4]. One of the most common side effects negatively impacting survivors’ quality of life is cancer-related fatigue (CRF), which develops during active cancer due to the disease process and/or its treatment and can persist long after therapy completion. The National Comprehensive Cancer Network (NCCN) defines CRF as a “distressing, persistent, and subjective sense of physical, emotional and/or cognitive tiredness or exhaustion not associated with recent activity and interfering with functioning due to a patient’s cancer or cancer treatment” [5]. One study found that fatigue was the symptom that had the strongest negative impact on patients’ quality of life, even more than pain, nausea, and vomiting [6]. However, fatigue in cancer survivors has previously been underreported, underdiagnosed, and undertreated [5]. This could be attributed to a lack of clear recommendations for screening, lack of a standardized screening tool, and differences between how long after treatment fatigue should be categorized as CRF [7]. A recent meta-analysis of 84 research studies including 144,813 subjects estimated that the pooled prevalence of CRF was 52%, but other estimates of CRF in the survivorship population are reported to be as high as 85% [8].

Sexual and gender minority (SGM) populations include individuals whose sexual orientation, gender identity or expression, or reproductive development is characterized by nonbinary constructs of sexual orientation, gender, and/or sex. Existing evidence suggests a higher incidence of certain cancers, such as breast, prostate, and colorectal cancers, in the SGM population [9]. There is limited research on SGM cancer survivor experiences due to failure of health systems and national cancer registries to reliably collect sexual orientation and gender identity data, rendering this group nearly invisible in large data sets [9]. Most CRF studies do not report how sex and gender data are collected and use these terms interchangeably or inconsistently [5]. SGM cancer survivors, compared to heterosexual counterparts, often experience higher levels of distress, depression, and impairment in activities of daily living, in addition to lower quality of life and self-rated health [10–12]. While there is some data on health experiences of SGM individuals impacted by cancer, there is a paucity of data specifically examining fatigue. One study was found on this topic, in which ten SGM cancer patients were compared to eight non-SGM controls [13]. In this small group, SGM patients reported greater anxiety and depression, while cisgender-heterosexual patients reported higher levels of CRF and pain intensity. Additionally, SGM patients reported greater experiences of social isolation, whereas cisgender-heterosexual patients reported more emotional support and companionship [13].

Unique challenges faced by the SGM population that may contribute to these disparities include minority stress, discrimination, unmet needs related to SGM identity, and limited support [13–18]. A systematic review examining patient perspectives of cancer care among SGM individuals revealed that most patients expressed dissatisfaction with their cancer care and experienced discrimination and inadequate support throughout their treatment [18]. These disparities contributed to heightened levels of anxiety, stress, depression, and negative perceptions of healthcare providers [18]. Minority stress and discrimination could be posited as pathways to elevated CRF, given that sustained vigilance and ongoing psychological burden likely amplify fatigue. Prior work has shown that cisgender sexual minorities and Black adults report more daytime sleepiness than cisgender-heterosexual and White individuals [19]. Additionally, higher levels of discrimination and depressive symptoms among sexual minority and Black adults were associated with increased daytime sleepiness [19]. A systematic review of health outcomes of SGM patients after cancer found a paucity of studies describing the experiences of transgender/gender diverse (TGD) persons and none which reported on experiences of intersex persons [15]. TGD survivors report transphobia and discrimination in the oncology care setting, including invalidation of or a lack of consideration for their TGD identity and the absence of inclusive supportive care resources [14]. TGD cancer survivors have also described anxiety when attending appointments or avoiding necessary follow up care, which could contribute to worse outcomes [14, 15].

The current study aims to compare fatigue and mental health outcomes between SGM and cisgender-heterosexual cancer survivors utilizing the *All of Us* research database. Understanding the burden of these concerns within the SGM population would allow clinicians to implement and test the impact of evidence-based interventions to decrease fatigue (such as physical activity, sleep hygiene and psychotherapy) to improve symptoms and functioning in SGM cancer survivors, or to develop SGM-tailored interventions if necessary [5, 20].

## Material and methods

This study is a secondary analysis of the *All of Us* Research dataset, accessed using the Researcher Workbench (researchallofus.org). University of Cincinnati has signed a Data Use and Registration Agreement to use the *All of Us* Researcher Workbench. Data is stored in the secure *All of Us* Researcher Workbench platform. This study is approved as Non-Human Subjects Research by the University of Cincinnati Institutional Review Board (IRB # 2025-0273).

### All of us research program database

Access to the secure *All of Us* Researcher Workbench is available after affiliated institutions sign a Data Use and Registration Agreement. Researchers create an account, including setting up two-factor authentication, verifying identity through Login.gov or ID.me, completing the *All of Us* Responsible Conduct of Research training, and signing a Data User Code of Conduct, which prohibits any re-identification of *All of Us* respondents.

All data available to researchers has had direct identifiers removed and has been modified to minimize re-identification risks. This comprises removing all explicit electronic health record (EHR) identifiers, participant free-text responses, geolocation data smaller than U.S. three-digit zip code, living situations, race and ethnicity subcategories, active-duty military status, cause of death, and diagnosis codes potentially subject to public knowledge. Select demographic fields are generalized, such as income, age if greater than 89, and subcategories of race and ethnicity. All dates (as day of year ranging from one to 366) are systematically shifted backwards by a random number between 1 and 365.

### Respondents

National Institutes of Health’s *All of Us* Research Program (controlled tier version seven) data, which contains de-identified electronic health records of approximately 290,000 individuals, was utilized in this analysis. Eligible respondents are individuals who are able to provide informed consent and agree to participate in the program, including permission for linkage of survey responses with electronic health record data such as physical measurements, diagnoses, and medications. Respondents are recruited through healthcare provider organizations, community engagement partners, and direct volunteer enrollment. Because enrollment is voluntary, the cohort is designed to be large and diverse but should not be interpreted as nationally representative. Compared to the nationally representative National Health and Nutrition Examination Survey (NHANES), *All of Us* respondents are older, less likely to be White and non-Hispanic, have more years of education, and are more likely to have chronic medical conditions [21].

In the context of this study, the quality of the data is limited by their origin in the medical record or as survey responses. Medical record-derived data are transformed into the Observational Medical Outcomes Partnership Common Data Model in which diagnoses, procedures, medications, laboratory values, and physical measurements are mapped to standardized vocabularies where possible. As with other medical record-based studies, data quality may be limited by healthcare utilization, coding practices, and incomplete capture of care rendered outside of contributing health systems [21].

Respondents with a diagnosis represented under the Systematized Nomenclature of Medicine Clinical Terms (SNOMED) code for “malignant neoplastic disease” (363346000) who also completed the “Overall Health” survey questions were included for analysis. Initially, 49,181 respondents were identified from the *All of Us* dataset. Respondents whose only malignant neoplasm was nonmelanoma skin cancer (NMSC) (*n* = 8419) were identified using natural language processing of diagnosis names and excluded from this study because NMSC are typically indolent and rarely require systemic therapy, making their survivorship course fundamentally different from other malignancies [22]. Complete case analysis was employed; 4078 respondents with missing demographic variables (sex, gender, race, ethnicity, age) were excluded. This study followed the Strengthening of the Reporting of Observational Studies in Epidemiology (STROBE) reporting guidelines.

### Measurements

Information collected included respondent sexual orientation, gender, race, ethnicity, age at first cancer diagnosis, and presence of diagnostic codes for anxiety (SNOMED 197480006 which maps to International Classification of Diseases [ICD]−10 F41 [23]. Other anxiety disorders); major depressive disorder (SNOMED 370143000 which maps to ICD-10 F32.0–9 Major depressive disorder); or fatigue (SNOMED 84229001 which maps to ICD-10 R53 Malaise and fatigue). Thus, “fatigue” includes diagnostic codes for cancer-related fatigue, weakness, chronic fatigue, and other fatigue. Outcomes included data from surveys assessing fatigue and mental health.

To compare the psychosocial experience of people based on demographic characteristics, several surveys collected by *All of Us* were selected. The *All of Us* program uses the Patient-Reported Outcomes Measurement Information System (PROMIS) Global Health survey to assess overall mental, social, and physical health [24]. These constructs were assessed using the following questions and Likert scales: “In the past 7 days, how would you rate your fatigue?” (*none, mild, moderate, severe, very severe*) and “In general, how would you rate your mental health, including your mood and your ability to think?” (poor, fair, good, very good, excellent). The PROMIS Global Health fatigue item is structurally similar to the clinically used One-item Fatigue Scale, as both assess self-reported fatigue severity using a brief ordinal response scale. However, the PROMIS item is administered within a broader health questionaire rather than as a standalone fatigue assessment, and responses may therefore be influenced by that context. Clinically, the One-item Fatigue scale, is used extensively, and has proven to be a fast, accurate way of screening patients for fatigue [25].

#### Minority groups

Responses to assigned sex at birth, gender identity, and sexual orientation were used to identify SGM individuals. Survey responses used to identify SGM respondents include: (1) A gender identity of “nonbinary,” “transgender,” “genderqueer,” “genderfluid,” “gender variant,” “two-spirit,” “none of these fully describe me, and I want to specify;” (2) Sex assigned at birth of “intersex” or “none of these fully describe me;” (3) Sexual orientation of “gay,” “lesbian,” “bisexual,” “queer,” “polysexual,” “omnisexual,” “sapiosexual,” “pansexual,” “asexual,” “two-spirit,” “mostly straight, but sometimes attracted to people of your own sex,” or “I mean something else.” Additionally, respondents with a current gender identity different from their sex assigned at birth were included as SGM individuals. Participants selecting "other" for gender identity or sexual orientation were given the option to provide a free-text response. These responses were manually reviewed and used to verify classification. Free-text entries were audited to ensure appropriate categorization and participants were classified as SGM only when the response indicated a sexual or gender minority identity. Respondents who declined to provide their race or ethnicity were excluded. Respondents who responded with a race other than “White” or an ethnicity other than “Not Hispanic or Latino” were identified as racial or ethnic minorities.

#### History of anxiety, depression, fatigue

To account for pre-existing mental health conditions and complaints that could confound results, we controlled for anxiety, depression, or fatigue diagnostic codes first recorded more than 12 months before the initial cancer diagnosis. This was determined by comparing the month of the first condition diagnosis with the month of the first cancer diagnosis. ICD-10 codes for depression and anxiety have high specificity but low sensitivity [26–28]. Of note, fatigue diagnostic codes have not been formally validated against a clinical reference standard, and definitions of fatigue vary in database research [26].

### Statistical analysis

Data searches, cohort building, and analysis solely took place on the Researcher Workbench using SAS® Viya Analytics Pro. Variables with *p* < 0.01 in univariable analyses were considered statistically significant and examined further. Frequencies and valid percentages were calculated. Survey responses were dichotomized consistent with methods in prior literature [29, 30]. Although dichotomizing ordinal scales reduces data granularity and study power, we chose this approach because we consider it to identify a clinically meaningful distinction; it also aligns with the National Comprehensive Cancer Network’s binary definition of cancer-related fatigue as greater than three on a ten-point scale (corresponding to “Moderate” or “Severe”) [5, 31]. For the fatigue item in the *All of Us* overall health survey, “Low” corresponds to “none” or “mild” and “High” corresponds to “Moderate,” “Severe,” or “Very Severe.” The scale is reversed for mental health; “Poor” corresponds to “Poor” or “Fair” and “Good” corresponds to “Good,” “Very Good,” or “Excellent.”

Unadjusted odds ratios were first computed to evaluate the relationship between fatigue or mental health survey responses and patient characteristics including: SGM status, racial or ethnic minority status, anxiety or depression (pre- or post-cancer), and fatigue (pre- or post-cancer). Multivariable logistic regression was performed with SGM status as the primary independent variable of interest to estimate the odds of low ratings of mental health or high levels of fatigue (taking note of the reversed coding as above) adjusting for preexisting anxiety or depression (versus never), subsequent anxiety or depression (versus never), preexisting fatigue (versus never), subsequent fatigue (versus never), age at first cancer (continuous, per year), and years of cancer survivorship (continuous, per year). Though highly heterogenous, we collapsed SGM subgroups to maintain ≥10 events per variable [32].

Of note, logistic regression can only determine odds ratios; these values should not be interpreted as risk ratios [33]. This distinction is of particular importance when the outcome is common as in the current study; odds ratios are known to overestimate risk or prevalence ratios in this situation and risk ratios must be directly estimated via models such as log-binomial regression or modified Poisson regression with robust variance [33]. As this is a secondary analysis focusing on the experiences of a relatively small subpopulation which uses a different outcome measure than the NCCN-recommended definition of CRF, logistic regression remains valid as a means to assess the direction and significance of associations with high fatigue with the caveat that our reported odds ratios likely overestimate risk ratios.

## Results

Demographic characteristics of the 36,684 cancer survivors included are summarized in Table 1. The mean age of the respondents in our dataset was 65.5 years and most (62%, *n* = 22,744) participated in *All of Us* five or more years after their first cancer diagnosis. Although most of the respondents in the dataset were White cisgender-heterosexual individuals, 6.6% (*n* = 2422) of the study population were SGM individuals, which is higher than the U.S. national average of 5.5%, and certainly higher than would be expected given our mean age of 65.5 years and that less than 3% of Americans 50 and older identify as SGM [34]. Additionally, 0.4% of our study population identified as TGD, which aligns with national estimates that 0.4% of those 35 to 64 years old identify as TGD and 0.3% of those 65 and older identify as TGD [34]. Among SGM survivors, 33% had moderate fatigue, 10% had severe fatigue, and 4% had very severe fatigue.

**Table 1:** Characteristics of cancer survivors in the database

Variable	*n* (%)
**Age at first cancer diagnosis (mean and 95% CI) **	58.5 (58.3–58.6)
**Age (mean and 95% CI)**	65.5 (65.3–65.6)
**Survivorship years (mean and 95% CI) **	7.3 (7.2–7.3)
**Gender identity**	
Cisgender	36,531 (99.6%)
Transgender, non-binary, or other gender	153 (0.4%)
Transgender	44 (0.1%)
Non-binary	81 (0.2%)
Other gender	71 (0.2%)
**Sexual orientation**	
Heterosexual	34,318 (93.6%)
Not heterosexual	2366 (6.4%)
Gay/Lesbian	1153 (3.1%)
Bisexual	917 (2.5%)
Other sexual minority	296 (0.8%)
**Sexual or gender minority**	
No	34,262 (93.4%)
Yes	2422 (6.6%)
**Race and ethnicity **	
White, non-Hispanic	28,188 (76.8%)
Racial or ethnic minority	8496 (23.2%)
**Anxiety or depression diagnosis **	
None	22,324 (60.9%)
Before cancer	4735 (12.9%)
After cancer	9625 (26.2%)
**Fatigue diagnosis**	
None	24,395 (66.5%)
Before cancer	2332 (6.4%)
After cancer	9957 (27.1%)
**Mental health**^a^	
Poor	4813 (13.1%)
Good	31,871 (86.9%)
**Fatigue**^b^	
Low	21,792 (59.4%)
High	14,892 (40.6%)

^a^For mental health, “Poor” corresponds to “Poor” or “Fair” and “Good” corresponds to “Good,” “Very Good,” or “Excellent”

^b^For fatigue, “Low” corresponds to “None” or “Mild” and “High” corresponds to “Moderate,” “Severe,” or “Very Severe”

In univariable analyses, SGM survivors had 1.62-fold higher odds of high fatigue than cisgender-heterosexual survivors (OR = 1.62, 95% CI [1.45, 1.81], *p* < 0.001 (see Table 2)). Pre-cancer anxiety/depression (aOR = 3.70, 95% CI [3.37, 4.07]*, p* < 0.001) and post-cancer anxiety/depression (aOR = 2.68, 95% CI [2.47, 2.90], *p* < 0.001) exerted the strongest association with fatigue (see Table 2). Adjusting for premorbid and comorbid diagnoses of anxiety, depression, and fatigue, SGM cancer survivors are more likely to report high levels of fatigue compared to cisgender-heterosexual cancer survivors.

**Table 2:** Counts and logistic regression for self-rated fatigue in *n* = 36,684 cancer survivors completing the survey

Variable (category)	Low fatigue^a^	High fatigue^a^	Univariable OR (95% CI)	Multivariable OR (95% CI)
**Gender**				
Cisgender-Heterosexual	30,851 (93.8%)	3415 (90.3%)	Ref	Ref
Sexual or Gender Minority	2050 (6.2%)	368 (9.7%)	1.62 (1.45–1.81) ***	1.33 (1.17, 1.51) ***
**Anxiety/Depression**^b^				
No anxiety/Depression	20,738 (63.0%)	582 (41.9%)	Ref	Ref
Pre-cancer Anxiety/Depression	3977 (12.1%)	757 (20.0%)	2.45 (2.29–2.73) ***	1.90 (1.71, 2.12) ***
Post-cancer Anxiety/Depression	8191 (24.9%)	1439 (38.1%)	2.30 (2.14–2.47) ***	2.00 (1.84, 2.18) ***
**Fatigue**^b^				
No fatigue	22,414 (68.1%)	1989 (52.6%)	Ref	Ref
Pre-cancer Fatigue	1966 (6.0%)	374 (9.9%)	2.14 (1.92–2.40)***	1.80 (1.58, 2.06) ***
Post-cancer Fatigue	8526 (25.9%)	1,415 (37.5%)	1.87 (1.75–2.00)***	1.82 (1.67, 1.98) ***
**Age at first cancer **(continuous, per year)	–	–	0.96 (0.96, 0.97)***	0.97 (0.97,0.98) ***
**Survivorship years** (continuous, per year)	–	–	0.92 (0.91, 0.93)***	0.94 (0.93, 0.95) ***

**p < 0.01; ***p < 0.001

^a^“Fatigue” as an outcome was identified with survey responses. “Low” corresponds to “None” or “Mild” and “High” corresponds to “Moderate,” “Severe,” or “Very Severe”

^b^“Anxiety,” “Depression,” and “Fatigue” as covariates were identified using diagnostic codes in the participant’s electronic medical record

For mental health variables, univariable analyses showed that SGM survivors have more than twice the odds of self-reported poor mental health compared with cisgender-heterosexual survivors (OR = 2.25; 95% CI [2.05, 2.47], *p* < 0.001) (see Table 3). Neither pre-cancer fatigue (aOR = 0.93, 95% CI [0.81, 1.06], *p* = 0.29) nor post-cancer fatigue (aOR = 1.07, 95% CI [0.99, 1.16], *p* = 0.08) significantly predicted poor mental health after adjustment. Older age (aOR per year = 0.970, 95% CI [0.968, 0.973], *p* < 0.001) and longer survivorship (aOR per year = 0.960, 95% CI [0.953, 0.966], *p* < 0.001) were both associated with better self-ratings of mental health. Adjusting for premorbid and comorbid diagnoses of anxiety, depression, and fatigue, self-reported mental health is more likely to be “poor” or “fair” among SGM cancer survivors compared to cisgender-heterosexual cancer survivors.

**Table 3:** Counts and logistic regression for self-rated mental health in *n* = 36,684 cancer survivors completing the survey

Variable (category)	Poor mental health^a^	Good mental health^a^	Univariable OR (95% CI)	Multivariable OR (95% CI)
**Gender**				
Cisgender-Heterosexual	4225 (87.9%)	30,046 (94.3%)	Ref	Ref
Sexual or Gender Minority	579 (12.1%)	1834 (5.7%)	2.25 (2.05–2.47) ***	1.83 (1.64–2.03) ***
**Anxiety/Depression**^a^				
No anxiety/Depression	1780 (37.0%)	20,561 (64.5%)	Ref	Ref
Pre-cancer Anxiety/Depression	1,173 (24.4%)	3541 (11.1%)	3.83 (3.55–4.13) ***	3.70 (3.37–4.07) ***
Post-cancer Anxiety/Depression	1860 (38.6%)	7769 (24.4%)	2.76 (2.59–2.96) ***	2.68 (2.47–2.90) ***
**Fatigue**^b^				
No fatigue	2910 (60.5%)	21,495 (67.4%)	Ref	Ref
Pre-cancer fatigue	377 (7.8%)	1954 (6.1%)	1.43 (1.28–1.59) ***	0.93 (0.81–1.06)
Post-cancer fatigue	1526 (31.7%)	8422 (26.4%)	1.34 (1.26–1.43) ***	1.07 (0.99–1.16)
**Age at first cancer** (continuous, per year)	–	–	0.96 (0.96–0.97) ***	0.97 (0.97–0.97) ***
**Survivorship years** (continuous, per year)	–	–	0.94 (0.94–0.95) ***	0.96 (0.95–0.97) ***

**p < 0.01; ***p < 0.001

^a^“Mental Health” as an outcome was identified with self-reported survey responses. “Poor” corresponds to “Poor” or “Fair” and “Good” corresponds to “Good,” “Very Good,” or “Excellent”

^b^“Anxiety,” “Depression,” and “Fatigue” as covariates were identified using diagnostic codes in the participant’s electronic medical record

## Discussion

This study reveals significant disparities in fatigue and mental health among SGM cancer survivors, even after adjusting for clinical factors. In this study, nearly half (47%) of SGM survivors experienced moderate or severe fatigue. In addition, SGM survivors experienced disproportionately higher odds of high fatigue compared to cisgender-heterosexual survivors [34]. These findings describe yet another burden experienced by individuals in the SGM population impacting their survivorship.

This study documents differences in fatigue and mental health between SGM patients and cisgender-heterosexual patients. Due to the observational nature and absence of explanatory analyses, our results are not able to suggest underlying mechanisms. Prior evidence suggests that mechanisms such as systematic inflammation, dysregulation of the hypothalamic–pituitary–adrenal axis, and minority stress may contribute to disparities in the SGM population [35, 36]. However, these constructs were not directly measured or analyzed in this study. Fatigue did not significantly predict poor mental health in the adjusted model, suggesting that while fatigue and mental health are correlated, distinct factors may be potent drivers of mental health and fatigue. For instance, a systematic review of patient reported outcomes among patients receiving cancer care found that SGM patients experienced discrimination, disparities, and inadequate resources when navigating care [18]. These encounters can lead to higher levels of anxiety, depression, and stress, and perhaps subsequent fatigue. A better understanding of the factors contributing to these disparities is needed before specific clinical interventions can be proposed. Understanding how mental health contributes to fatigue in SGM survivors across treatment phases warrants further investigation with longitudinal studies examining symptom trajectories.

Depression has been found to be a contributing factor of fatigue [8]. Similarly, our findings suggest that pre- and post-cancer anxiety/depression may influence fatigue among SGM cancer survivors. Current practices for addressing and treating fatigue include physical activity, psychosocial interventions, bright white light therapy, acupuncture, nutrition consultation, and pharmacologic interventions, but do not explicitly target anxiety/depression [37, 38]. In our study, SGM survivors experienced more than twice the odds of reporting poor mental health compared to cisgender-heterosexual survivors, consistent with previous literature documenting elevated psychological distress, higher rates of depression, and greater difficulties and distress in relationships [10]. Yang et al. found that SGM cancer patients reported greater cancer-related care concerns, more difficulty seeking help during treatment, and higher levels of mental and emotional distress [39]. These findings call for psychosocial interventions tailored to SGM cancer survivors to target these increased disparities. Disparities may vary across SGM subgroups and cancer types, as most prior work is specifically focused on the impact of breast cancer and prostate cancer and may not be generalizable to other diagnoses [15].

In addition to mental health interventions explicitly targeting anxiety/depression, SGM individuals may benefit from fatigue-specific interventions delivered by providers within settings that foster social safety and allow survivors to lower their level of threat-vigilance, which likely interferes with healing and may itself contribute to symptoms [40]. Cancer prevention and screening interventions tailored to the SGM population have been found to be more acceptable and feasible, suggesting SGM-tailored fatigue interventions may also be feasible and more acceptable [41]. Further, gendered cancer care environments can cause discomfort and disengagement among TGD patients [42]. Understanding how experiences of discomfort or discrimination during cancer treatment correlate with fatigue, and how clinician education on best practices in affirming care influences patient experiences and outcomes, is important. Patient navigator programs may offer a promising solution to improving patient experience and trust, and thus patient outcomes. In other contexts, navigator SGM training was found to improve cultural competency and increase comfort among SGM population [43, 44]. Therefore, employing SGM navigators into patient care programs could not only influence patient experiences and improve access to care by overcoming social vulnerability, but also indirectly have a protective effect on survivors’ symptoms.

Given the higher burden of fatigue among SGM cancer survivors, cancer care providers should screen for and address fatigue and mental health needs of SGM individuals impacted by cancer before, during, and after treatment. Outcomes for SGM survivors could be improved by routine collection of sexual orientation and gender identity data collection in cancer databases, oncology, and primary care settings. This data should pair with the integration of patient-reported outcomes measures on fatigue and mental health. Embedding SGM psycho-oncology referral pathways and piloting SGM-tailored CRF interventions that explicitly address minority stress and discrimination-related distress such as exercise, psychotherapy, and peer support can target underlying mechanisms that may impact fatigue. Further education for SGM-serving primary care clinicians practicing outside of cancer care settings on the care needs of survivors can ensure needs are addressed across the care continuum. Collectively, these strategies are essential to advancing equitable, comprehensive survivorship care and reducing fatigue burden among SGM cancer survivors.

### Limitations and future directions

While our study benefits from a large, diverse sample, limitations include reliance on self-reported data and lack of granularity in cancer type and treatment history. While the sample is relatively diverse with respect to SGM status, *All of Us* is a volunteer cohort with known selection biases and complex weighting issues. Similarity in overall SGM proportion is not sufficient to claim representativeness of U.S. SGM cancer survivors. Additionally, findings may not generalize to SGM survivors who are not engaged with large health systems or research programs. Potential selection effects from sampling such as self-selection, higher digital literacy, and differential survivorship could bias fatigue and mental health estimates. Prior literature has established that living with intersectional identities is not equivalent to self-reporting those identities in health care research surveys. Likewise, we cannot assume that SGM individuals consistently disclose these identities within All of Us surveys or health records.

The survey item for fatigue in *All of Us* differs from that used by the NCCN despite the fact that the fatigue survey question in the *All of Us* survey is similar to the One-item Fatigue scale that is widely used clinically. The One-Item Fatigue Scale has not been studied as thoroughly as the Functional Assessment of Chronic Illness Therapy–Fatigue (FACIT-F), which demonstrates robust psychometric properties for identifying CRF [45]. Additionally, our inclusion of survivors who responded to the *All of Us* survey more than five years beyond their initial cancer diagnosis does not align with the NCCN definition of CRF. Since 62% of the respondents participated more than 5 years after diagnosis, we use the terminology “fatigue” not CRF. We acknowledge there can be other reasons for fatigue than cancer, such as unidentified comorbidities, and the side effects of treatment; particularly for those who report fatigue 5 years post-diagnosis.

Dichotomizing survey responses limits our study power and results in loss of data detail. Given the high prevalence of fatigue in this cohort, odds ratios derived from logistic regression overestimate the risk ratio and must be interpreted strictly as odds ratios [33]. Diagnostic codes for anxiety, depression, and fatigue have low sensitivity, and definitions of “fatigue” as administrative codes vary in published literature, so our identification of participants with these conditions in this sample is limited [26–28]. Our results are also limited in that we were unable to disaggregate subgroup-specific experiences (i.e., TGD vs cisgender sexual minorities, monosexual vs plurisexual individuals, or racialized SGM survivors vs White SGM survivors) given limited sample sizes. Better collection of sexual orientation and gender identity data in cancer registries could improve our ability to better understand additional vulnerabilities and opportunities within SGM subgroups.

## Conclusion

SGM cancer survivors are more likely to report worse fatigue and mental health compared to cisgender-heterosexual cancer survivors. This study describes the unique burden of fatigue experienced by SGM cancer survivors and the need for tailored interventions and longitudinal studies across treatment phases. Future interventional studies are needed to mitigate the unique fatigue and mental health needs of this population. Cancer care providers should address fatigue and mental health needs of SGM individuals impacted by cancer before, during, and after treatment to minimize the impact on their daily life. Addressing these disparities can improve quality of life and long-term survivorship outcomes.

## Data Availability

This study used data from the All of Us Research Program’s Controlled Tier Dataset version seven, available to authorized users on the Researcher Workbench.
